# Effects of Soil Physico-Chemical Properties on Plant Species Diversity Along an Elevation Gradient Over Alpine Grassland on the Qinghai-Tibetan Plateau, China

**DOI:** 10.3389/fpls.2022.822268

**Published:** 2022-02-04

**Authors:** Wangya Han, Li Chen, Xukun Su, Dan Liu, Tiantian Jin, Songlin Shi, Tao Li, Guohua Liu

**Affiliations:** ^1^Jiangsu Key Laboratory of Agricultural Meteorology, Institute of Ecology, School of Applied Meteorology, Nanjing University of Information Science and Technology, Nanjing, China; ^2^State Key Laboratory of Urban and Regional Ecology, Research Center for Eco-Environmental Sciences, Chinese Academy of Sciences, Beijing, China; ^3^Torch High Technology Industry Development Center, Ministry of Science and Technology, Beijing, China; ^4^College of Resources and Environment, University of Chinese Academy of Sciences, Beijing, China; ^5^Institute of Qinghai-Tibetan Plateau, Southwest Minzu University, Chengdu, China; ^6^China Institute of Water Resources and Hydropower Research, Beijing, China; ^7^College of Tourism and Urban-Rural Planning, Chengdu University of Technology, Chengdu, China

**Keywords:** alpine grassland, plant community, species diversity, soil physico-chemical properties, elevation gradient

## Abstract

Elevation gradient can reflect the effects of soil physico-chemical properties on plant species diversity. Alpine grassland on the QTP has suffered from a serious decline in plant species diversity. In this study, we investigated 112 sites recording plant community characteristics and collecting soil samples along an elevation gradient (3,500–5,200 m asl) in alpine meadow on the QTP. We analyzed the effects of soil physico-chemical properties on plant species composition and diversity by canonical ordination and spatial regression along an elevation gradient. The results showed that species richness of the overall plant communities decreased with the increasing elevation, and the Simpson dissimilarity index (*β_*sim*_*) had a maximum at low elevation (3,500–4,000 m) with the value of 0.37. Soil available nitrogen content was the primary soil parameter affecting plant species composition and diversity in alpine grassland. The effect of soil available nitrogen content on plant species richness varied at different elevations. For Gramineae plants (G), plant species richness declined with the increase in soil available nitrogen content at low elevation (3,500–4,000 m), but rose at middle elevation (4,000–4,500 m). Soil available nitrogen content had a more significant limiting effect on species richness at high elevation (>4,500 m). These findings increase our understanding about the drivers of plant species diversity changes in alpine grassland on the QTP, and will provide insights into grassland restoration and sustainable management.

## Introduction

Plant species diversity is the crucial factor that has important impact on plant community structure and types, focusing on species coexistence in alpine grassland ecosystem (Xu et al., 2011; Dong et al., 2019). The study of plant species diversity is the basis for evaluating ecosystem functions (Wang et al., 2012). There has been a strong relationship between plant species diversity and key ecosystem functions including plant production and soil organic matters (Hooper et al., 2012; Zhu et al., 2020). It has been reported that soil physico-chemical properties lead to changes in plant species diversity. The relationship between plant diversity and soil physico-chemical properties is vulnerable to ecological contexts (Grace et al., 2016; Zhu et al., 2020), therefore, it is imperative to evaluate the effect of soil physico-chemical properties on plant species diversity along environmental gradients.

Some studies have documented that plant species diversity is affected by environmental parameters, such as temperature, humidity, solar radiation, and soil nutrients (Dong et al., 2019). Most studies address temperature as the dominant factor of species diversity (Barbarossa et al., 2021; Lai et al., 2021). A series of studies have analyzed the relationship between plant species diversity and environmental parameters. The relationships between plant species diversity and soil physico-chemical properties have been analyzed in terms of topographic parameters (Li X. et al., 2018), such as geological structure and lithology (Do Carmo and Jacobi, 2016; Hanaka et al., 2019). Natural gradient studies have been conducted to explore the mechanisms of species diversity changes, and elevation gradient is one of the important research contents because elevation affects the distribution of moisture and energy (Li X. et al., 2018). In addition, the elevation gradient plays an important role in exploring how species diversity affected by environmental parameters (Supriya et al., 2019; Lai et al., 2021). Although a variety of elevational species diversity patterns have been detected, few studies have considered the responses of species diversity to environmental variation among different plant community types, analyzing the community dissimilarity along the elevation gradient. The community dissimilarity can be quantified by β-diversity (Haider et al., 2018), so the diversity variation should be carried out combining both α-diversity and β-diversity of plant communities.

The QTP has complex topographic features with contiguous mountains and hills. As the highest and most extensive highland in the world, QTP has extensive species diversification and alpine endemic species (Yu et al., 2019). Moreover, the warming rate on the QTP has been almost twice that of general global warming over recent decades (Kuang and Jiao, 2016; Guo et al., 2018). Due to spatially complex topography configurations and climate changes, the QTP has suffered from serious decline of species diversity (Li et al., 2019). Alpine meadow is the main ecosystem on the QTP (Liu et al., 2018), accounting for approximately 35% of the total plateau area (Xu et al., 2011). It has important ecological and socio-economic roles, such as storing carbon, altering biodiversity by evolving grass species that shape environments (Gieselman et al., 2013; Dong et al., 2019). The species diversity of alpine meadow on the QTP has attracted the attention of numerous researchers (Ebersbach et al., 2017; Yu et al., 2019). However, the responses of species diversity to soil physico-chemical properties distinguishing between different community types along an elevation gradient were rarely conducted.

In this study, we focused on the relationship between plant species diversity and soil physico-chemical properties. We explored the effect of soil physico-chemical properties on α and β diversity of plant species along an elevation gradient, providing information for restoration and sustainable development of alpine meadow. The specific objectives were to (1) identify changes of plant species diversity and soil physico-chemical properties along the elevation gradient; (2) clarify which soil physico-chemical properties affect plant species composition and diversity, and (3) elucidate the effects of key soil parameters on species diversity at different elevations.

## Materials and Methods

### Study Area

**FIGURE 1:**
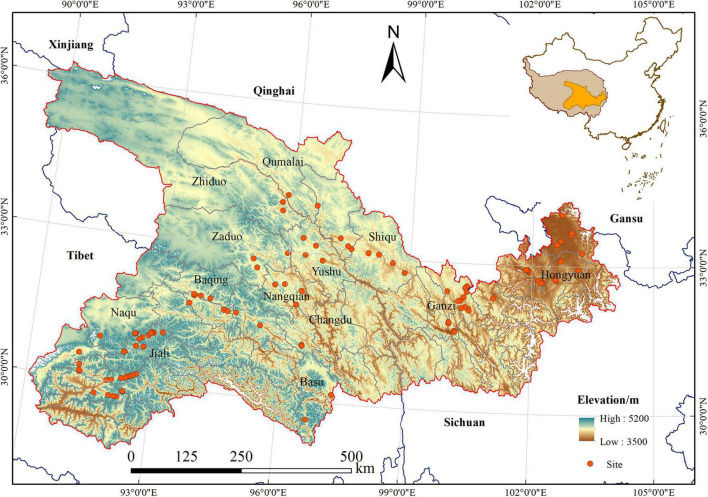
Location of the study area.

The study area is located in the mid-eastern part of the QTP (89°30′∼104° 20′ E, 28° 35′∼36° 24′ N) (Figure 1), and includes Naqu, Jiali, Changdu, Basu, and Baqing Counties in the Tibetan autonomous prefecture, Qumalai, Zhiduo, Zaduo, and Nangqian Counties in Qinghai Province, and Ruoergai, Aba, Ganzi, and Hongyuan Counties in Sichuan Province. It covers an area of 5.3 × 10^5^ km^2^ and accounts for nearly 25% of the QTP. The elevation ranges from 3,500 to 5,200 m above sea level, with geographical characteristics of mountains and valley plains. The study area is in the temperate semi-arid monsoon climate zone. In the past 35 years (1980–2015), the mean annual temperature ranges from −9.5°C to 11.4°C, and the multi-year average precipitation is from 152 to 1,218 mm. The vegetation type is mainly alpine meadow. The soil types are mainly felt soil, meadow soil, and frigid calcic soil, followed by dark felt soil, permafrost soil, and dark brown soil (Chinese Soil Taxonomy Research Group, 1995).

### Sampling Design

The plant community was sampled during the growing season between July and August, 2017 and 2018. We selected 112 field sites [low elevation (3,500–4,000 m): 45, middle elevation (4,000–4,500 m): 38, high elevation (>4,500 m): 29] with representative alpine meadow in space-for-time substitution (Niu et al., 2019), ranging from 3,500 to 5,200 m. These sites were under similar site condition, with the middle slope position and slope degree ranging from 12° to 18°. The grazing intensity is moderate grazing, according to the classification of grazing intensity from Sun et al. (2019). They contained natural vegetation and were homogeneous sites within the study area (Figure 1). Each site covered an area of 900 m^2^ (30 m × 30 m). We established five 1 m × 1 m quadrats, which were placed at the four corners and at the center of the site. Within each quadrat, we measured the cover, height, and density of every vascular plant species, and we recorded latitude, longitude, elevation, and topographic slope using a handheld global positioning system (GPS) device. Five soil samples were collected at a depth of 0–20 cm in each quadrat.

### Data Collection and *A*nalysis

#### Vegetation Data Collection

We recorded the coverage of all plants as the fractional vegetation cover, and the species names and abundance of each vascular plant species. The height of each species was evaluated by randomly measuring five individual plants (Han W. Y. et al., 2019). We also recorded the coverage and frequency of each species in each quadrat. The nomenclature follows Flora of China^1^. The importance values (IVs) of the species were calculated as follows (Li C. et al., 2018):


$${\text{P}}_{i}=\left(\frac{{\text{C}}_{i}}{\sum_{i}^{S}{\text{C}}_{i}}+\frac{{\text{H}}_{i}}{\sum_{i}^{S}{\text{H}}_{i}}+\frac{{\text{F}}_{i}}{\sum_{i}^{S}{\text{F}}_{i}}\right)/3\times 100$$

where *P*_*i*_ is the importance value (IVs) of species *i*; *C*_*i*_ is the coverage of species *i*; *H*_*i*_ is the height of species *i*; and *F*_*i*_ is the frequency of species *i*.

We used α diversity and β diversity to describe species diversity. The α diversity indices used were the species richness index (*R*), the Shannon-Wiener index (*H’*), and Pielou’s evenness index (*J*). For β diversity, the Sørenson’s dissimilarity index (*β_*sor*_*) was used to measure the overall diversity based on presence-absence species data and represented diversity among communities, and *β_*sor*_* was partitioned into the Simpson dissimilarity index (*β_*sim*_*) and the nestedness resultant dissimilarity index (*β_*sne*_*). The diversity indices were calculated as follows (Wu and Ding, 2020; Lai et al., 2021):


R=S

$$H^{\prime }=-\sum_{i=1}^{s}\left(P_{i}\,{\mathrm{lnP}}_{i}\right)$$

$$J=-\sum_{i=1}^{s}\left(P_{i}\,{\mathrm{lnP}}_{i}\right)/\mathrm{lnS}$$

$$\beta_{s\,o\,r}=\frac{a+b}{2\,t+a+b}$$

$$\beta_{s\,i\,m}=\frac{m\,i\,n\,\left(a,b\right)}{a+m\,i\,n\,\left(a,b\right)}$$

$$\beta_{n\,e\,s}=\beta_{s\,o\,r}-\beta_{s\,i\,m}$$

where *S* is the number of species in the plot; *t* is the number of species shared by two communities or plots; *a* and *b* represent the total number of species in two communities or plots, respectively.

#### Soil Physico-Chemistry Analysis

Soil samples were collected at a depth of 0–20 cm from the surface in each field quadrat. A total of five soil samples were collected from each quadrat and the soil parameters were the average of the five samples. The soil samples were air-dried and sieved, and the soil moisture content and soil bulk density were determined by the oven drying method according to Thomasson (2010). Soil pH was measured using a pH meter and a 1:1 water/soil suspension, and soil total carbon content and soil total nitrogen content were measured using the flash dynamic combustion method and a gas chromatographic separation and thermal conductivity detection system (vario EL cube Elementar, Germany), respectively (Feng et al., 2017). Soil organic carbon content was determined by the dichromate oxidation method and soil available nitrogen content was measured by the alkali-hydrolysis reduction diffusion method (Bao, 2000). Soil available phosphorus content was extracted using the ammonium bicarbonate method and soil available potassium content was extracted using the acetamide extraction method (Zhang et al., 2018).

#### Climate and Topography Data

The climate parameters were interpolated into climate surface data using thin plate smoothing splines method by the ANUSPLIN software package (version 4.4) (Hutchinson and Xu, 2013; Xu and Hutchinson, 2013). The output statistics are interpreted with the partial spline model for *N* (*N* = 135) observed data values *z*_*i*_ given by,


$$z_{i}=f\left(x_{i}\right)+b^{T}y_{i}+e_{i}\left(i=1,\ldots ,N\right)$$

where each *x*_*i*_ (temperature and precipitation data) is a d-dimensional vector of spline independent variables, *f* is an unknown smooth function of the *x*_*i*_, each *y*_*i*_ is a p-dimensional vector of coefficients of the *y*_*i*_ (elevation data) and each *e*_*i*_ is an independent (Hutchinson and Xu, 2013). The temperature and precipitation data were derived from the national weather stations data for 1982–2015 (freely available at the website: http://data.cma.cn/site). The elevation data was derived from digital elevation models at a 1 km resolution and were obtained by resampling the data from CGIAR-CSI (freely available at the website: http://srtm.csi.cgiar.org).

### Statistical Analysis

We used the *vegan*, *betapart*, and *cluster* packages in R version 3.5.0 to analyze species composition and diversity in the different communities. The plant communities were classified by hierarchical clustering using Ward’s method and the Bray-Curtis distance. We used non-metric multidimensional scaling (NMDS) analysis to show the species cluster characteristics of the different plant communities.

Statistical analyses of soil parameters were performed using SPSS Statistics software (version 20). One-way analysis of variance (ANOVA) was used to compare significant differences among the soil physico-chemical properties of the different plant communities. A paired significance analysis was undertaken using the Scheffé test when the variation was significant (*p* < 0.05). The correlations between species diversity (species richness, Shannon-Wiener index, and evenness) and soil physico-chemical properties were calculated using Pearson’s correlation coefficient by the function cor and psych package in R version 3.5.0. Stepwise multiple linear regression was used to screen the variables affecting species richness. The vegan package in R version 3.5.0 was used to perform a canonical correspondence analysis (CCA) to further confirm the relationships between species data and soil parameters in the different plant communities. A Mantel test (999 permutations) was used to test the significance of the relationships and the linear model (LM) was used to explore the significant relationship between species richness and its primary factor. The partial correlation analysis was used to eliminate the interference of other soil parameters to soil available nitrogen content.

## Results

### Changes in Plant Species Composition and Diversity Along the Elevation Gradient

There were 369 herbaceous plant species belonging to 174 genera and 49 families. Families with larger IVs (>1) included Cyperaceae, Gramineae, Rosaceae, Compositae, Ranunculaceae, Polygonaceae, Leguminosae, Gentianaceae, Scrophulariaceae, Euphorbiaceae, Labiaceae, Primulaceae, and Umbelliferae in descending order. The plant communities were classified into six clusters by the cluster analysis, which was based on Bray-Curtis distance matrices (Supplementary Figure 1). After combining the cluster results with ecological background information and other related studies (Antony et al., 2016; Zhang et al., 2018), the classifications were as follows: *Kobresia pygmaea* (Kp), *Kobresia pygmaea* + miscellaneous plants (KpM); *Kobresia setchwanensis* (Ks), *Carex moorcroftii* (Cm), miscellaneous plants (M); and Gramineae plants (G). The dominant species in the different plant communities were characterized by their IVs (Table 1).

**TABLE 1:** The dominance of species selected by IVs across six plant community types.

Plant community types	Species names	Family	IV
Kp	*Kobresia pygmaea*	Cyperaceae	9.472
	*Carex moorcroftii*	Cyperaceae	3.750
	*Potentilla saundersiana*	Rosaceae	2.984
	*Leontopodium leontopodioides*	Compositae	1.823
	*Stipa capillata*	Gramineae	1.150
	*Astragalus propinquus*	Leguminosae	1.062
	*Kobresia tibetica*	Cyperaceae	0.833
	*Kobresia setschwanensis*	Cyperaceae	0.817
	*Poa crymophila*	Gramineae	0.780
	*Taraxacum mongolicum*	Compositae	0.738
	*Anaphalis sinica*	Compositae	0.728
	*Oxytropis alpina*	Leguminosae	0.702
	*Polygonum macrophyllum*	Polygonaceae	0.656
	*Potentilla anserina*	Rosaceae	0.584
	*Aster himalaicus*	Compositae	0.544
KpM	*Kobresia pygmaea*	Cyperaceae	3.090
	*Potentilla saundersiana*	Rosaceae	1.524
	*Carex moorcroftii*	Cyperaceae	1.022
	*Leontopodium leontopodioides*	Compositae	0.936
	*Salix cupularis*	Salicaceae	0.638
	*Polygonum viviparum*	Polygonaceae	0.522
Ks	*Kobresia setschwanensis*	Cyperaceae	1.607
	*Potentilla saundersiana*	Rosaceae	0.817
	*Kobresia pygmaea*	Cyperaceae	0.676
	*Carex moorcroftii*	Cyperaceae	0.572
	*Elymus nutans*	Gramineae	0.503
Cm	*Carex moorcroftii*	Cyperaceae	2.292
	*Kobresia pygmaea*	Cyperaceae	1.302
	*Potentilla saundersiana*	Rosaceae	0.780
	*Polygonum viviparum*	Polygonaceae	0.686
	*Kobresia setschwanensis*	Cyperaceae	0.660
	*Leontopodium leontopodioides*	Compositae	0.570
	*Potentilla anserina*	Rosaceae	0.542
M	*Kobresia pygmaea*	Cyperaceae	1.813
	*Carex moorcroftii*	Cyperaceae	1.624
	*Kobresia setschwanensis*	Cyperaceae	1.589
	*Potentilla saundersiana*	Rosaceae	1.299
	*Leontopodium leontopodioides*	Compositae	0.768
	*Polygonum viviparum*	Polygonaceae	0.670
	*Kobresia tibetica*	Cyperaceae	0.657
G	*Elymus nutans*	Gramineae	0.762
	*Poa crymophila*	Gramineae	0.530

*Kp, Kobresia pygmaea; KpM, Kobresia pygmaea + Miscellaneous plants; Ks, Kobresia setchwanensis; Cm, Carex moorcroftii; M, Miscellaneous plants; G, Gramineae plants.*

**FIGURE 2:**
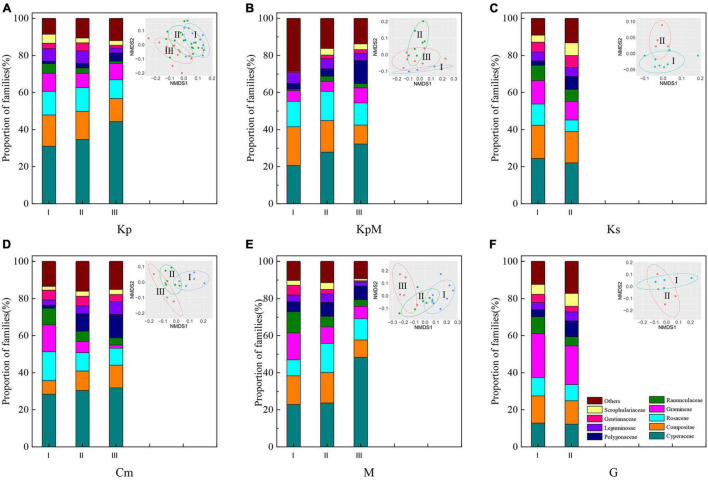
Composition of the plant communities at the family level and non-metric multidimensional scaling analysis (NMDS) of plant composition based on the Bray-Curtis distance. **(A)** Kp, *Kobresia pygmaea*; **(B)** KpM, *Kobresia pygmaea*+ Miscellaneous plants; **(C)** Ks, *Kobresia setchwanensis*; **(D)** Cm, *Carex moorcroftii*; **(E)** M, Miscellaneous plants; **(F)** G, Gramineae plants.

The relative proportions of the families in six plant communities were distinct (Figure 2). Kp, KpM, Cm, and M were dominated by the Cyperaceae; the Cyperaceae and Compositae proportions were similar for Ks; and Gramineae (22.66%) was predominant in G. The NMDS plot (Figure 2) shows the species composition characteristics of the different plant communities along coordinate 1 and coordinate 2 of an elevation gradient (I, low elevation; II, middle elevation; III, high elevation). The species composition profile of Kp at high elevation was different from the other elevation classes. The species composition profiles at low and middle elevations for all community types except Ks tended to group together (Figure 2). The Cyperaceae proportions rose in all the community types other than Ks and G with increasing elevation gradient. For Kp and KpM, the Compositae proportions decreased as elevation increased, but the Compositae proportion in Cm obviously increased with an average growth rate of 30.44%. For M, the Gramineae proportion decreased as elevation increased and reached a minimum of 6.78% at high elevation (Figure 2).

**FIGURE 3:**
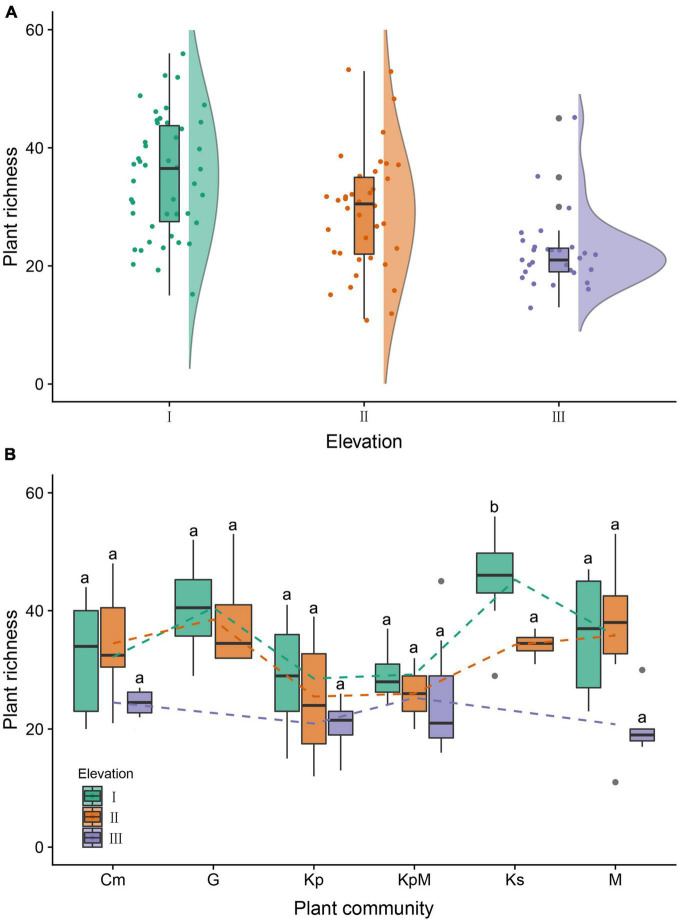
Box and raincloud plots of plant richness along the elevation gradient. **(A)** Plant species richness as influenced by elevation. I: low elevation (3,500–4,000 m), II: middle elevation (4,000–4,500 m), III: high elevation (>4,500 m). **(B)** Plant species richness as influenced by elevation under different plant communities. Kp, *Kobresia pygmaea*; KpM, *Kobresia pygmaea*+ Miscellaneous plants; Ks, *Kobresia setchwanensis*; Cm, *Carex moorcroftii*; M, Miscellaneous plants; G, Gramineae plants. Bars with the same letter within the same elevation class denote means that are not significantly different at *p* ≤ 0.05. Error bars depict standard error of the means.

The *α-*diversity characteristics of plant species (species richness, Shannon-Wiener index, and Pielou’s evenness index) showed a certain regularity along the elevation gradient (Table 2). The species richness of the overall plant communities showed an obviously decreasing trend as the elevation rose, and followed the order of low elevation (35.24) > middle elevation (29.45) > high elevation (22.24) (Figure 3A). At low elevation, species richness in Ks was significantly higher than in Kp, KpM, Cm, M, and G. The species richness for Kp and KpM dropped gradually as the elevation increased, and species richness for Cm and M rose at first and then decreased as the elevation increased (Figure 3B). The Shannon-Wiener index for the overall communities showed a downward trend as elevation increased and the Pielou’s evenness index reached a maximum at middle elevation. There was a difference in *β-*diversity of the overall plant communities along the elevation gradient, but the differences among elevation classes were not significant (Table 3). Simpson dissimilarity index (*β_*sim*_*) at low elevation was 0.37, as the largest one among elevation classes. Nestedness resultant dissimilarity index (*β_*sne*_*) continued to increase with the rise of elevation. For different community types, the highest Sørenson’s dissimilarity index (*β_*sor*_*) was between KpM and Ks with a value of 0.68, followed by the index between KpM and G (0.63) (Supplementary Table 1).

**TABLE 2:** Characteristics of species *α-*diversity at different elevation classes.

Diversity characteristics	Plant community type	Low elevation	Middle elevation	High elevation
Richness index	Kp	28.56 ± 4.83	25.50 ± 3.85	20.93 ± 3.32
	KpM	29.25 ± 5.56	26.00 ± 4.43	25.29 ± 10.72
	Ks	45.25 ± 8.20	34.25 ± 2.50	–
	Cm	32.20 ± 5.45	34.50 ± 4.65	24.50 ± 2.38
	M	35.78 ± 5.73	35.83 ± 5.22	20.80 ± 5.26
	G	40.50 ± 9.61	38.50 ± 9.95	–
Shannon-Wiener index	Kp	2.87 ± 0.34	2.71 ± 0.38	2.52 ± 0.21
	KpM	2.72 ± 0.16	2.74 ± 0.19	2.69 ± 0.36
	Ks	3.34 ± 0.31	3.09 ± 0.13	–
	Cm	2.97 ± 0.35	3.03 ± 0.31	2.75 ± 0.15
	M	3.03 ± 0.39	3.04 ± 0.57	2.53 ± 0.35
	G	3.24 ± 0.36	3.24 ± 0.27	–
Pielou’s evenness index	Kp	0.87 ± 0.03	0.85 ± 0.04	0.83 ± 0.04
	KpM	0.81 ± 0.03	0.85 ± 0.03	0.85 ± 0.03
	Ks	0.88 ± 0.04	0.88 ± 0.02	–
	Cm	0.86 ± 0.02	0.86 ± 0.02	0.86 ± 0.03
	M	0.85 ± 0.04	0.87 ± 0.03	0.84 ± 0.05
	G	0.88 ± 0.04	0.89 ± 0.02	–

*Kp, Kobresia pygmaea; KpM, Kobresia pygmaea + Miscellaneous plants; Ks, Kobresia setchwanensis; Cm, Carex moorcroftii; M, Miscellaneous plants; G, Gramineae plants.*

**TABLE 3:** The ANOVA result of *β-*diversity along the elevation gradient.

Elevation	*β_*sor*_*	*β_*sim*_*	*β_*sne*_*	*β_*ratio*_*
Low elevation	0.46 ± 0.17*^ns^*	0.37 ± 0.14*^ns^*	0.08 ± 0.05*^ns^*	0.82 ± 0.10*^ns^*
Middle elevation	0.45 ± 0.09*^ns^*	0.35 ± 0.10*^ns^*	0.10 ± 0.07*^ns^*	0.77 ± 0.14*^ns^*
High elevation	0.47 ± 0.06*^ns^*	0.34 ± 0.11*^ns^*	0.13 ± 0.07*^ns^*	0.72 ± 0.20*^ns^*

*β_sor_, Sørenson’s dissimilarity index; β_sim_, Simpson dissimilarity index; β_sne_, nestedness resultant dissimilarity index; β_ratio_, the ratio of β_sim_ to β_sor_. ns, no significant differences (P > 0.05).*

### Variation in Soil Physical and Chemical Properties Along the Elevation Gradient

Elevation had a significant impact (*p* < 0.05) on soil physico-chemical properties, such as soil moisture content, soil pH, and soil available nitrogen content, in all the plant communities (Table 4). However, the soil physico-chemical properties were not significantly influenced (*p* < 0.05) by plant community type at the same elevation class. Soil moisture content for Kp was significantly greater by 1.8 times at high elevation compared to the low and middle elevations. Soil pH significantly varied (*p* < 0.05) along the elevation gradient for Kp in the order high elevation (5.53) < low elevation (5.89) < middle elevation (6.67). Soil available nitrogen content increased significantly (*p* < 0.05) from middle elevation to high elevation for KpM and Cm, and was 8.5 and 2.6 times greater at high elevation than at middle elevation for KpM and Cm, respectively. A significant (*p* < 0.05) increase in soil available nitrogen content for Kp was observed at high elevation, with an average of 246.45 mg kg^–1^ at high elevation, 56.98 mg kg^–1^ at low elevation, and 43.67 mg kg^–1^ at middle elevation. Soil available nitrogen content was significantly greater (*p* < 0.05) by six times at high elevation compared to low elevation for M (Supplementary Figure 2).

**TABLE 4:** Soil physico-chemical properties as influenced significantly by elevation for different plant communities.

Plant community type	Soil physico-chemical property	Low elevation	Middle elevation	High elevation
Kp	SMC	24.79 ± 7.95a	22.38 ± 7.15a	62.41 ± 15.71b
	SpH	5.89 ± 0.53a	6.67 ± 0.64b	5.53 ± 0.26a
	SAN	56.98 ± 15.95a	43.57 ± 16.06a	246.45 ± 56.21b
KpM	SAN	43.11 ± 12.36a	45.44 ± 12.36a	384.65 ± 62.15b
Cm	SAN	57.87 ± 4.49a	61.82 ± 12.50a	161.59 ± 43.27b
M	SAN	59.11 ± 28.69a	47.74 ± 15.55a	313.59 ± 47.86b

**Analyses of variance (*p*-values)**				
**Variable**	**Elevation class**	**Community type**	**Elevation × Community**	

SMC	*p* < 0.01	0.196	*p* < 0.05	
SpH	*p* < 0.05	0.27	0.059	
SAN	*p* < 0.001	0.788	0.469	

*Kp, Kobresia pygmaea; KpM, Kobresia pygmaea + Miscellaneous plants; Cm, Carex moorcroftii; M, Miscellaneous plants; SMC, soil moisture content; SpH, soil pH; SAN, soil available nitrogen content. Letters indicate significant differences among elevation classes (p < 0.05).*

### Soil Physico-Chemical Properties Effects on Plant Diversity

**FIGURE 4:**
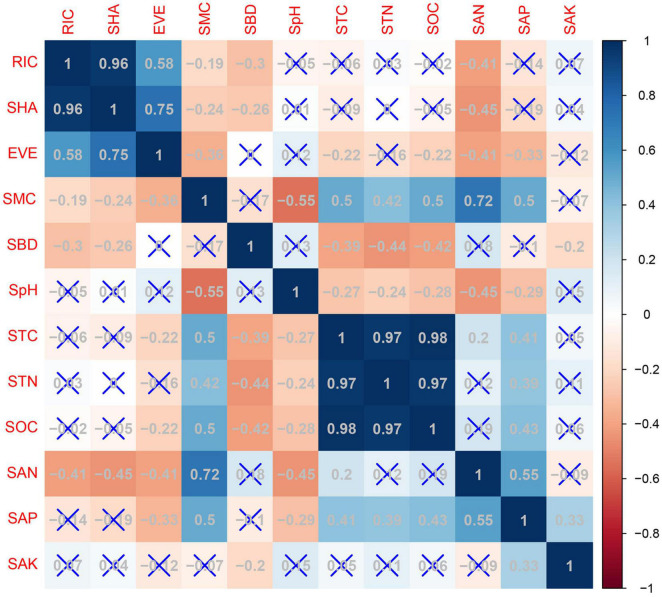
Pearson’s correlations between species diversity and soil parameters (RIC, species richness; SHA, Shannon-Wiener index; EVE, species evenness; SMC, soil moisture content; SBD, soil bulk density; SpH, soil pH; STC, soil total carbon content; STN, soil total nitrogen content; SOC, soil organic carbon content; SAN, soil available nitrogen content; SAP, soil available phosphorus content; SAK, soil available potassium content).

Figure 4 shows the Pearson’s correlations between species diversity and soil physico-chemical properties. Species richness was significantly negatively correlated with soil available nitrogen content (*r* = −0.41, *p* < 0.01), soil bulk density (*r* = −0.29, *p* < 0.01), and soil moisture content (*r* = −0.19, *p* < 0.05). Species richness was significantly positive correlated with the Shannon-Wiener index (*r* = 0.96, *p* < 0.01), and with species evenness (*r* = 0.58, *p* < 0.01). Therefore, species richness was chosen as the species variable. A stepwise multiple linear regression was used to determine the effect of plant species richness on the explanatory variables, such as soil physico-chemical properties and community types (Type 1: Kp, *Kobresia pygmaea*; Type 2: Cm, *Carex moorcroftii*; Type 3: Ks, *Kobresia setchwanensis*; Type 4: KpM, *Kobresia pygmaea* + Miscellaneous plants; Type 5: M, Miscellaneous plants) (Table 5). The ANOVA analysis indicated that soil available nitrogen content, Type 2, and soil pH had significant impacts (*p* < 0.01) on species richness. Soil available nitrogen content contributed the most (46.1%) to the variance in species richness explained by the regression model (*R*^2^ = 0.35), followed by Type 2 (23.5%), soil pH (14.1%), community type 5 (9.9%), and soil bulk density (6.3%).

**TABLE 5:** Results of a stepwise multiple linear regression of species richness against explanatory variables.

Variable	Estimate (standard error)	ANOVA		Relative importance
		*F*-value	Pr(>F)	
SAN	0.65	22.32	***	46.1%
Type 2	0.17	18.81	***	23.5%
SpH	−0.40	16.25	***	14.0%
Type 5	−0.36	14.47	***	10.0%
SBD	−0.49	12.10	***	6.3%

SAN, soil available nitrogen content; Type, plant community type (Type 1: Kp, Kobresia pygmaea; Type 2: Cm, Carex moorcroftii; Type 3: Ks, Kobresia setchwanensis; Type 4: KpM, Kobresia pygmaea+ Miscellaneous plants; Type 5: M, Miscellaneous plants); SpH, soil pH; SBD, soil bulk density. *** Shows the significant correlation at 0.001 levels.

**FIGURE 5:**
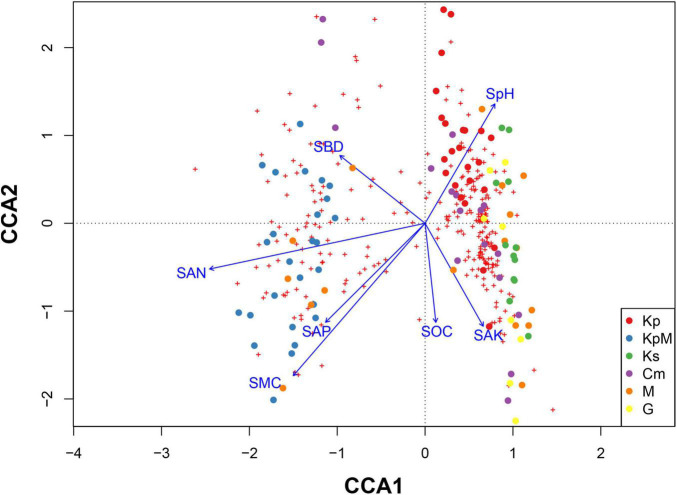
Canonical correspondence analysis showing the relationships between species and soil parameters across different plant communities. Kp, *Kobresia pygmaea*; KpM, *Kobresia pygmaea*+ Miscellaneous plants; Ks, *Kobresia setchwanensis*; Cm, *Carex moorcroftii*; M, Miscellaneous plants; G, Gramineae plants; SMC, soil moisture content; SBD, soil bulk density; SpH, soil pH; STC, soil total carbon content; STN, soil total nitrogen content; SOC, soil organic carbon content; SAN, soil available nitrogen content; SAP, soil available phosphorus content; SAK, soil available potassium content.

The CCA results indicated that soil available nitrogen content and soil moisture content were the most influential factors among the soil parameters because they drove changes in the composition and diversity of plant species. Plant species composition and diversity followed the first two axes of the ordination with soil parameters (Figure 5). The overall Mantel significance test was significant (*F* = 2.411, *p* < 0.001), and the significance test was also significant for the first axis (*F* = 7.107, *p* < 0.001) and the second axis (*F* = 2.555, *p* < 0.001). The first axis accounted for 42.11% of the total variation with an eigenvalue of 0.4261 and the second axis accounted for 15.14% with an eigenvalue of 0.1532. Soil available nitrogen content was strongly negatively correlated (−0.94) and soil bulk density was negatively correlated (−0.39) with the first axis. Soil moisture content had a strong negative correlation with the second axis (–0.76) and soil pH was positively correlated with the second axis (0.60).

**FIGURE 6:**
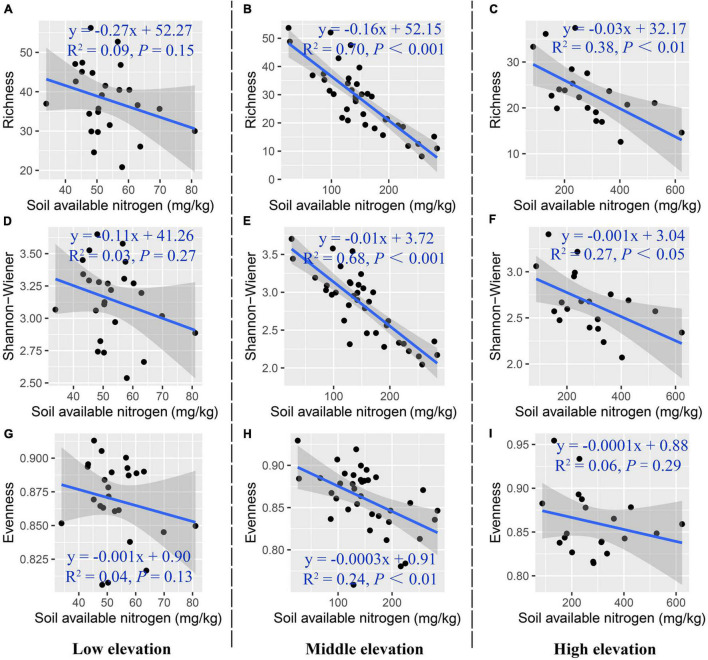
Partial correlation between plant species diversity and soil available nitrogen content along the elevation gradient. **(A–C)** Species richness, **(D–F)** Shannon-Wiener index, **(G–I)** species evenness.

Figure 6 shows the partial correlation coefficients between species diversity and soil available nitrogen content along an elevation gradient, regardless of soil moisture content. At low elevation, species diversity decreased insignificantly with the increase of soil available nitrogen content. At middle elevation, species diversity had a significant negative relationship with soil available nitrogen content [species richness (*R*^2^_*partial*_ = 0.70, *p*_*partial*_ < 0.001), Shannon-Wiener index (*R*^2^_*partial*_ = 0.68, *p*_*partial*_ < 0.001), evenness (*R*^2^_*partial*_ = 0.24, *p*_*partial*_ < 0.01)]. At high elevation, species richness decreased significantly with increasing soil available nitrogen content (*R*^2^_*partial*_ = 0.38, *p*_*partial*_ < 0.01), and Shannon-Wiener index had a significant negative relationship with soil available nitrogen content (*R*^2^_*partial*_ = 0.27, *p*_*partial*_ < 0.05).

## Discussion

### Effects of Soil Available Nitrogen Content on Species Composition and Diversity Along the Elevation Gradient

Elevation is a decisive factor influencing the distribution pattern of plant species because it leads to variations in hydrothermal conditions (Antony et al., 2016; Tesfaye et al., 2016). Our study showed that the species richness of all plant communities declined as elevation increased (Figure 3A). The species richness of Ks was significantly higher than other communities at low elevation, because Cyperaceae and Compositae had the similar proportions in Ks, with larger niche dimension (Xu et al., 2011). The Sørensen dissimilarity index (*β_*sor*_*) of the overall plant communities based on the species richness was smallest at middle elevation, with no obvious trend as the increasing elevation. Simpson’s dissimilarity index (*β_*sim*_*) as a component of *β-*diversity is not considered the influence of the richness gradient, and the nestedness resultant dissimilarity index (*β_*sne*_*) is based on subsets with lowest species richness (Baselga, 2010). *β_*sim*_* of the overall plant communities decreased and *β_*sne*_* increased gradually with the increasing elevation (Table 3). *β_*sor*_* between KpM and Ks was the largest at low elevation and middle elevation. It may be considered with physiologic limitation as *Kobresia setchwanensis* is hard to survive due to the low temperature at high elevation (Xie et al., 2014).

The conversion of soil available nitrogen is impeded due to the extreme climate conditions in alpine regions (Xu et al., 2011). Elevation is a restrictive factor for soil available nitrogen content. It influences the soil available nitrogen conversion because the hydrothermal conditions vary at different elevations. Plant growth in an alpine meadow ecosystem is strongly limited by available nitrogen (Jiang et al., 2017; Han Y.H. et al., 2019). The partial correlation coefficient between plant species richness and soil available nitrogen content was insignificant on the overall elevation, and there was a decrease in species richness of 0.916 with every 100 mg kg^–1^ increase in soil available nitrogen content. For different elevations, the relationships between species richness and soil available nitrogen content shown by the partial correlation regression tended to decrease insignificantly (Supplementary Figure 3).

Species richness was affected by soil available nitrogen content to different extents at the different elevations (Figure 6). Our study eliminated the redundancy effects of each factor by using stepwise multiple linear regression. The results showed that soil available nitrogen content explained the most variance (46.1%) in the regression model for species richness against the explanatory variables. The LM analysis, which controlled the elevation variable (Figure 6), showed that Cm species richness significantly increased [1.14 ± 0.73 (mg kg^–1^)^–1^] and the species richness of Ks significantly decreased [−0.47 ± 0.23 (mg kg^–1^)^–1^] with increasing soil available nitrogen content at low elevation. The species richness value for G had the highest intercept [2.13 ± 1.57 (mg kg^–1^)^–1^] at middle elevation. Furthermore, species richness decreased for all plant communities (Kp, KpM, Cm, and M) appearing at high elevation, but this was not obvious (Table 6).

**TABLE 6:** Intercepts for species richness response to soil available nitrogen content by the different plant communities along the elevation gradient.

Plant community type	Low elevation		Middle elevation		High elevation	
	Intercept	Standard error	Intercept	Standard error	Intercept	Standard error
Kp	0.260	0.153	0.280	0.126	−0.001	0.007
KpM	0.021	0.018	0.065	0.026	−0.030	0.015
Cm	1.142	0.732	0.461	0.310	−0.008	0.009
M	−0.145	0.044	−0.065	0.019	−0.005	0.009
Ks	−0.467	0.232	−0.367	0.281		
G	−0.311	0.270	2.131	1.579		

*Kp, Kobresia pygmaea; KpM, Kobresia pygmaea + Miscellaneous plants; Ks, Kobresia setchwanensis; Cm, Carex moorcroftii; M, Miscellaneous plants; G, Gramineae plants.*

### Effects of Climate Factors on Species Richness and Soil Available Nitrogen

In addition to the changes of hydrothermal conditions caused by elevation, the climatic background had impacts on species richness and soil available nitrogen content. The climate of the study area located in the southeast of the QTP is clearly warming and the rates of the temperature rise have been found to increase with increasing elevation (Song et al., 2014; Kuang and Jiao, 2016). The precipitation on the QTP is generally increasing, but annual precipitation in the study area shows an insignificant downward trend. The variation in precipitation does not have a uniform spatial distribution and the changes in precipitation are not as pronounced as the changes in temperature (Kuang and Jiao, 2016). It is widely acknowledged that climate factors are the main drivers of species richness patterns because they influence the effects of temperature on rates of species evolution or interactions between organisms (Kubota et al., 2015). Some studies have found that climate warming enhances the mineralization of soil organic matter, which contains most of the soil nitrogen (Kahmen et al., 2006; Manning et al., 2008). This mineralization leads to soil nutrient enrichment and the available nitrogen in soil increases (Klein et al., 2004). The multiple regression analysis of soil available nitrogen content against its influential factors indicated that temperature ranked next to elevation among the environmental factors at 8.4% (Supplementary Table 2). The increase in temperature will accelerate the decomposition of nitrogen in soil organic matter, which will affect soil available nitrogen content.

### Implications for Alpine Grassland Management

Analyzing the effects of soil physico-chemical properties on plant species composition and diversity within different plant communities along an elevation gradient helps explain species distribution patterns and the formation of communities. The resource utilization modes used by different plant species vary among community types. They depend on the characteristic differences among the niches utilized by the dominant species (Thakur and Wright, 2017). Nitrogen is the main limiting element on plant growth in alpine grassland ecosystems (Gao et al., 2013; Jiang et al., 2017), especially for Cyperaceae plants. Soil available nitrogen content increased along axis 1 from right to left, and KpM was mainly distributed across a spatial area with a high soil available nitrogen content (Figure 6). Climate warming causes plants to migrate from lower to higher altitudes, and it can also directly reduce plant diversity by replacing dominant and native species that have been disappeared due to habitat loss (Steinbauer et al., 2018; Niu et al., 2019). The result that nitrogen enrichment caused by soil warming reduces the loss of plant species diversity provides a theoretical foundation for scientific fertilization (Xu et al., 2011). The results can be used to provide guidance to the grassland management of different community types, improving grassland restoration programs and the construction of alpine grassland (Li X. et al., 2018).

## Conclusion

In this study, we divided plant communities into six types based on species importance values and analyzed how plant species composition/diversity was influenced by soil parameters along an elevation gradient. The results showed that species richness decreased as elevation increased for all plant communities, but the trend was not significant among community types. *β-*diversity of the overall plant communities varied insignificantly among different elevation classes. For different community types, the Sørenson’s dissimilarity index (*β_*sor*_*) between KpM and Ks was the largest (0.68). Soil available nitrogen content was the most important factor driving the changes in plant species composition and diversity among the soil parameters. Our results further indicated that the effect of soil available nitrogen content on plant species richness was influenced by elevation, and that the limiting effect of soil available nitrogen content was more obvious at high elevation. Furthermore, climate warming accelerated the decomposition of soil organic nitrogen and temperature was the most important environmental factor after elevation. Therefore, nitrogen mineralization caused by climate warming exacerbated the decline in species richness at high elevation. Our study explores the responses of plant species diversity to physico-chemical properties by distinguishing different community types along an elevation, and might provide detective meaning for sustainable management of alpine grassland.

## Data Availability Statement

The original contributions presented in the study are included in the article/Supplementary Material, further inquiries can be directed to the corresponding author/s.

## Author Contributions

WH and GL designed the study. LC and DL conducted the experiments and organized and analyzed the data. XS, TL, TJ, and SS participated in the field works. WH and LC led the writing of the manuscript. GL and XS modified manuscript structure. All authors contributed to the article and approved the submitted version.

## Conflict of Interest

The handling editor declared a shared affiliation, though no other collaboration, with several of the authors WH, LC, XS, TL, and GL at the time of the review. The remaining authors declare that the research was conducted in the absence of any commercial or financial relationships that could be construed as a potential conflict of interest.

## Publisher’s Note

All claims expressed in this article are solely those of the authors and do not necessarily represent those of their affiliated organizations, or those of the publisher, the editors and the reviewers. Any product that may be evaluated in this article, or claim that may be made by its manufacturer, is not guaranteed or endorsed by the publisher.

## Footnotes

1
foc.iplant.cn
