# Supplementary material for: Effects of Soil Physico-Chemical Properties on Plant Species Diversity Along an Elevation Gradient Over Alpine Grassland on the Qinghai-Tibetan Plateau, China
Source: Front Plant Sci. 2022 Feb 4;13:822268. doi: 10.3389/fpls.2022.822268 (PMC8854778; doi:10.3389/fpls.2022.822268)
Supplement: Supplementary file 1 [file Table_1.docx]

Supplementary Material

# Supplementary Tables

**Supplementary Table 1.** *β*-diversity of different plant communities along the elevation gradient.

| Plant community pair | low elevation | | | |  | middle elevation | | | |  | high elevation | | | |
| --- | --- | --- | --- | --- | --- | --- | --- | --- | --- | --- | --- | --- | --- | --- |
|  | *β_sor_* | *β_sim_* | *β_sne_* | *β_ratio_* |  | *β_sor_* | *β_sim_* | *β_sne_* | *β_ratio_* |  | *β_sor_* | *β_sim_* | *β_sne_* | *β_ratio_* |
| Kp-KpM | 0.49 | 0.29 | 0.20 | 0.59 |  | 0.35 | 0.26 | 0.09 | 0.74 |  | 0.46 | 0.43 | 0.03 | 0.93 |
| Kp-Ks | 0.38 | 0.33 | 0.05 | 0.87 |  | 0.51 | 0.25 | 0.26 | 0.49 |  |  |  |  |  |
| Kp-Cm | 0.39 | 0.35 | 0.04 | 0.90 |  | 0.35 | 0.22 | 0.14 | 0.61 |  | 0.58 | 0.44 | 0.13 | 0.77 |
| Kp-M | 0.37 | 0.33 | 0.04 | 0.88 |  | 0.39 | 0.28 | 0.11 | 0.71 |  | 0.38 | 0.20 | 0.18 | 0.52 |
| Kp-G | 0.31 | 0.27 | 0.04 | 0.86 |  | 0.49 | 0.29 | 0.21 | 0.58 |  |  |  |  |  |
| KpM-Ks | 0.74 | 0.59 | 0.14 | 0.81 |  | 0.63 | 0.52 | 0.11 | 0.82 |  |  |  |  |  |
| KpM-Cm | 0.65 | 0.54 | 0.10 | 0.84 |  | 0.48 | 0.45 | 0.03 | 0.94 |  | 0.47 | 0.26 | 0.21 | 0.55 |
| KpM-M | 0.74 | 0.61 | 0.13 | 0.82 |  | 0.47 | 0.45 | 0.02 | 0.96 |  | 0.47 | 0.27 | 0.20 | 0.57 |
| KpM-G | 0.69 | 0.59 | 0.09 | 0.86 |  | 0.57 | 0.48 | 0.09 | 0.84 |  |  |  |  |  |
| Ks-Cm | 0.37 | 0.28 | 0.10 | 0.74 |  | 0.40 | 0.27 | 0.14 | 0.66 |  |  |  |  |  |
| Ks-M | 0.22 | 0.21 | 0.01 | 0.97 |  | 0.54 | 0.42 | 0.12 | 0.78 |  |  |  |  |  |
| Ks-G | 0.35 | 0.24 | 0.10 | 0.71 |  | 0.40 | 0.36 | 0.04 | 0.90 |  |  |  |  |  |
| Cm-M | 0.39 | 0.30 | 0.09 | 0.77 |  | 0.31 | 0.30 | 0.01 | 0.96 |  | 0.47 | 0.46 | 0.01 | 0.98 |
| Cm-G | 0.39 | 0.39 | 0.00 | 1.00 |  | 0.37 | 0.29 | 0.09 | 0.77 |  |  |  |  |  |
| M-G | 0.35 | 0.26 | 0.09 | 0.73 |  | 0.46 | 0.37 | 0.09 | 0.81 |  |  |  |  |  |

Notes: *β_sor_*, Sørenson’s dissimilarity index; *β_sim_*, Simpson dissimilarity index; *β_sne_*, nestedness resultant dissimilarity index; *β_ratio_*, the ratio of *β_sim_* to *β_sor_*. Kp, *Kobresia pygmaea*; KpM, *Kobresia pygmaea* + Miscellaneous plants; Ks, *Kobresia setchwanensis*; Cm, *Carex moorcroftii*; M, Miscellaneous plants; G, Gramineae plants.

**Supplementary Table 2.** Results of a stepwise multiple linear regrassion of soil available nitrogen content against explanatory variables.

| Variable | ANOVA | | Importance |
| --- | --- | --- | --- |
|  | F-value | Pr(＞ F) |  |
| SMC | 120.97 | *** | 52.4% |
| Ele | 35.96 | *** | 11.8% |
| Tem | 33.13 | *** | 8.4% |
| SAP | 14.09 | *** | 3.2% |
| SBD | 4.84 | 0.03 | 1.0% |

Notes: SMC, soil moisture content; Ele, elevation; Tem, average annual temperature; SAP, soil available phosphorus content; SBD, soil bulk density.
