# Supplementary material for: Effects of Soil Physico-Chemical Properties on Plant Species Diversity Along an Elevation Gradient Over Alpine Grassland on the Qinghai-Tibetan Plateau, China
Source: Front Plant Sci. 2022 Feb 4;13:822268. doi: 10.3389/fpls.2022.822268 (PMC8854778; doi:10.3389/fpls.2022.822268)
Supplement: Supplementary file 2 [file Image_1.pdf]

Supplementary Material

1 Supplementary Figures

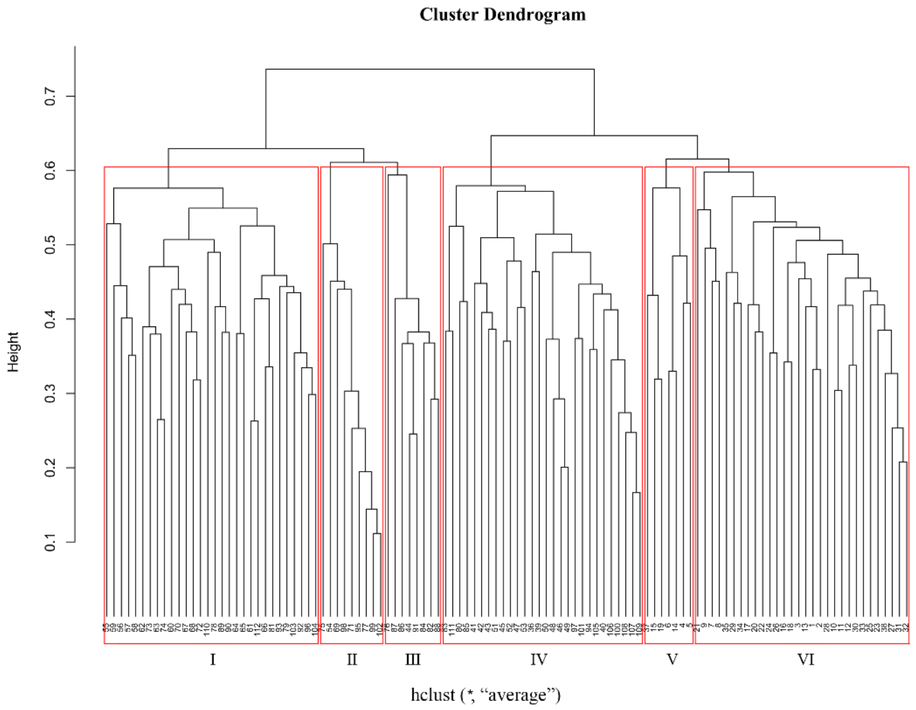

Supplementary Figure 1. Cluster analysis (Bray-Curtis distance) of plant communities. I:Cluster 1, II: Cluster 2, III: Cluster 3, IV: Cluster 4, V: Cluster 5, VI: Cluster 6.

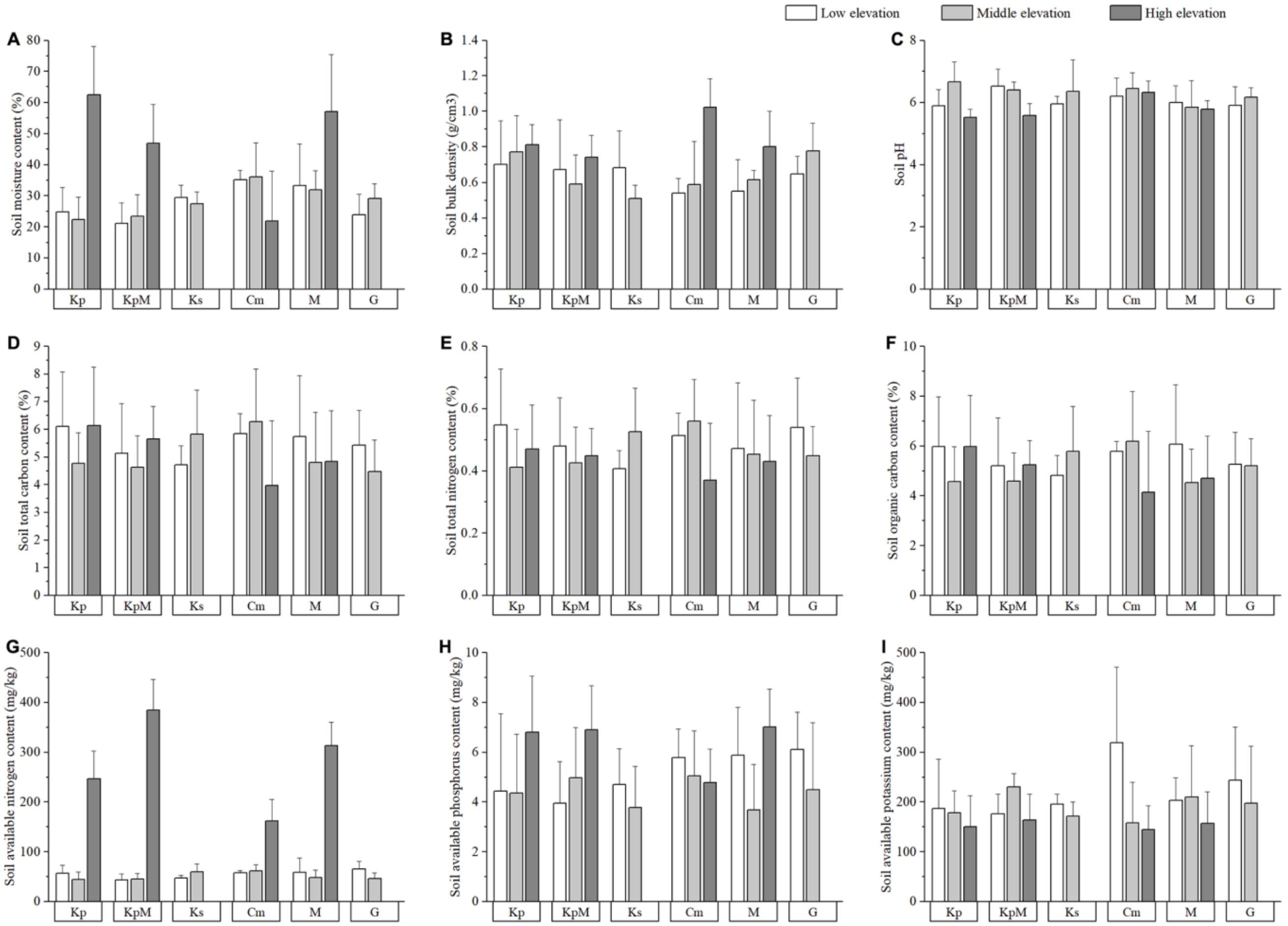

Supplementary Figure 2. Influence of elevation on the soil physico-chemical properties of the different plant communities. (A) Soil moisture content, (B) soil bulk density, (C) soil pH, (D) soil total carbon content, (E) soil total nitrogen content, (F) soil organic carbon content, (G) soil available nitrogen content, (H) soil available phosphorus content, (I) soil available potassium content. Kp: *Kobresia pygmaea*, KpM: *Kobresia pygmaea* + Miscellaneous plants, Ks: *Kobresia setchwanensis*, Cm: *Carex moorcroftii*, M: Miscellaneous plants, G: Gramineae plants.

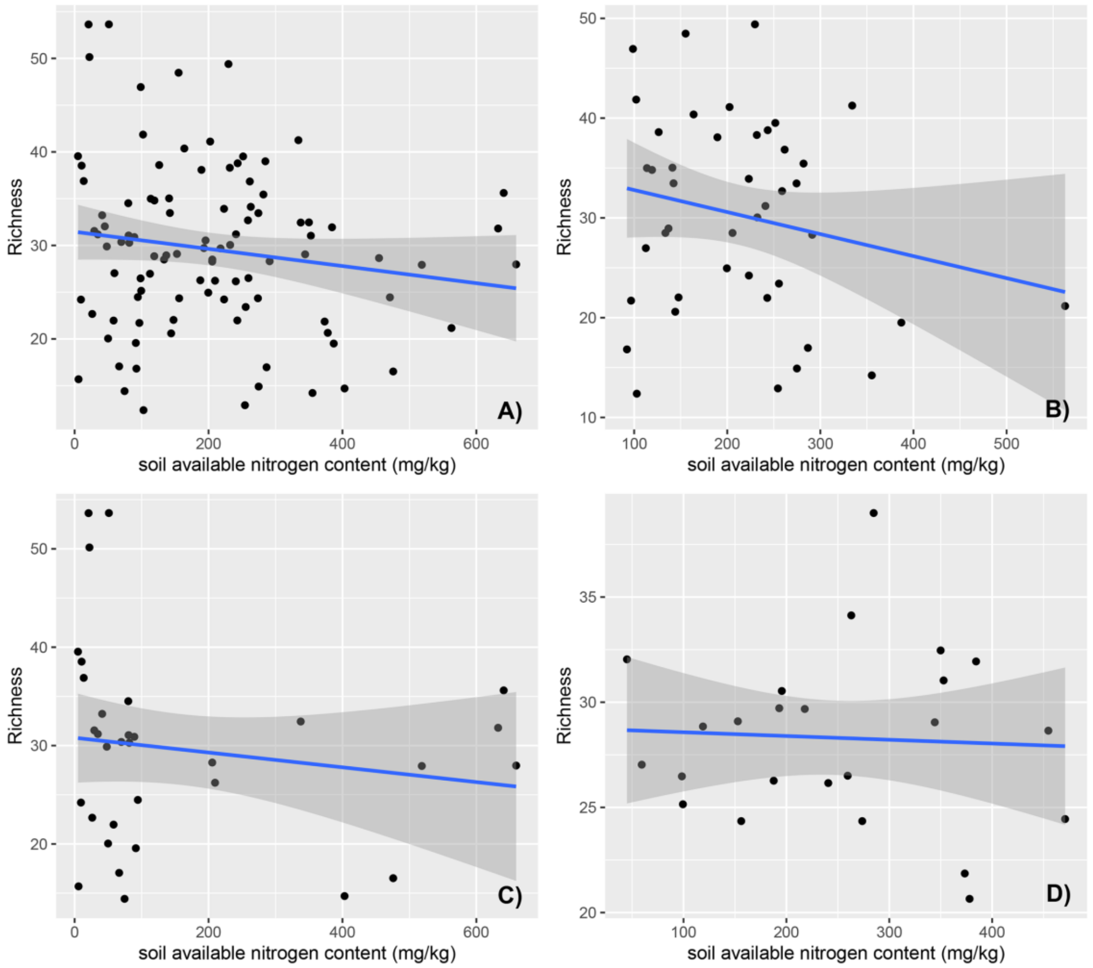

Supplementary Figure 3. Relationships between soil nitrogen content and plant species richness in partial correlation regression. (A) Overall elevation, (B) low elevation, (C) middle elevation, (D) high elevation.
